# 伴有SMARCA4缺失的非小细胞肺癌9例临床病理分析

**DOI:** 10.3779/j.issn.1009-3419.2022.102.27

**Published:** 2022-08-20

**Authors:** 汝楠 赵, 宜覃 邹, 鸿远 陈, 燕华 陈, 艳芳 刘, 妙侠 何

**Affiliations:** 1 200433 上海，海军军医大学第一附属医院/上海长海医院临床病理科 Department of Pathology, The First Affiliated Hospital of Naval Medical University/Shanghai Changhai Hospital, Shanghai 200433, China; 2 200433 上海，海军军医大学第一附属医院/上海长海医院呼吸内科 Department of Respiratory Medicine, The First Affiliated Hospital of Naval Medical University/Shanghai Changhai Hospital, Shanghai 200433, China

**Keywords:** SMARCA4, 肺肿瘤, 病理学特征, 诊断, SMARCA4, Lung neoplasms, Pathological features, Diagnosis

## Abstract

**背景与目的:**

SMARCA4缺失的非小细胞肺癌（SMARCA4-deficient non-small cell lung cancer, SMARCA4-dNSCLC）是一种罕见的原发于肺的恶性肿瘤。虽然2021版世界卫生组织（World Health Organization, WHO）肺肿瘤分类中并未将其单独列出，但这类肿瘤具有独特的形态学、免疫表型及分子遗传学特征。本研究通过探讨SMARCA4-dNSCLC的临床病理特征、免疫组化、诊断及鉴别诊断，以加深对该类肿瘤的认识。

**方法:**

收集2020年1月-2022年3月上海长海医院诊断的9例SMARCA4-dNSCLC的临床与影像学资料，通过组织学、免疫组化染色分析其临床病理特征并进行文献复习。

**结果:**

9例患者中位发病年龄65岁。6例男性均有吸烟史。肿瘤平均直径3.3 cm。6例转移。影像学提示为边界不清的浸润性肿块，3例伴胸膜侵犯。9例诊断为SMARCA4-dNSCLC，主要表现为3种病理形态，包括经典的肺腺癌、肺黏液腺癌及低分化癌。低分化肿瘤细胞上皮样、合体样或横纹肌样，胞质丰富，胞质可完全透明至嗜酸性，可见嗜酸性小球或小脓肿，呈实性片状，间质较多炎细胞及片状坏死。免疫组化显示SMARCA4均为阴性，8例细胞角蛋白（cytokeratin 5.2, CAM5.2）、细胞角蛋白7（cytokeratin 7, CK7）弥漫强阳性，P40均阴性，6例甲状腺转录因子1（thyroid transcription factor-1, TTF-1）阴性，2例部分阳性，1例阳性。

**结论:**

SMARCA4-dNSCLC是一类罕见的肺癌亚型，病理形态复杂多样，特征性的免疫组化表型可协助诊断。

## Abstract (en)

**Background and objective:**

SMARCA4-deficient non-small cell lung cancer (SMARCA4-dNSCLC) is a rare primary lung malignancy. These diseases are not listed separately in the 2021 World Health Organization (WHO) classification of lung neoplasms, but they have special morphological, immunophenotypic and molecular genetic characteristics. This study aims to improve understanding of SMARCA4-dNSCLC by discussing the clinicopathological features, diagonosis and differential diagnosis of the disease.

**Methods:**

The clinical and imaging data of 9 cases of SMARCA4-dNSCLC diagnosed in Shanghai Changhai Hospital from January 2020 to March 2022 were collected. The clinicopathological features were analyzed by histological and immunohistochemical staining, and the literature was reviewed.

**Results:**

The median age of 9 patients was 65 years old. Six men were smokers. The average maximum diameter of tumor was 3.3 cm. Six cases had been metastasized. The imaging showed that it was an infiltrating mass with unclear boundary and 3 cases invaded the pleura. Nine cases were diagnosed as SMARCA4-dNSCLC, which mainly showed three pathological forms including classic lung adenocarcinoma, mucinous adenocarcinoma and poorly differentiated carcinoma. Poorly differentiated tumor cells are epithelioid, syncytial or rhabdomyoid, the cytoplasm was rich, the cytoplasm could be completely transparent to eosinophilic, eosinophilic globules or small abscesses could be seen, showing solid flakes, with more inflammatory cells and flake necrosis in the stroma. Immunohistochemistry showed that SMARCA4 was negative in all cases and eight cases demonstrated cytokeratin 5.2 (CAM5.2) and cytokeratin 7 (CK7) was diffusely strongly positive, p40 was negative, thyroid transcription factor-1 (TTF-1) was negative in 6 cases, partially positive in 2 cases and diffusely positive in 1 case.

**Conclusion:**

SMARCA4-dNSCLC is a rare subtype of lung cancer with complex and diverse pathological morphology. The characteristic of immunohistochemical phenotype can assist in the diagnosis.

*SMARCA4*作为一种抑癌基因，位于19p13.2，编码BRG1蛋白，该蛋白是SWI/SNF染色质重塑复合体的重要组成亚基之一^[1]^。其中SWI/SNF复合体是转录和各种DNA修复的强大调控因子，并在细胞周期、细胞分化等过程中发挥重要作用。当SMARCA4蛋白失活，可导致SWI/SNF复合体中该亚基功能丧失并引起ATP供能中断，从而影响复合物重新定位或重塑核小体，由于无法接近需要转录的DNA区域，转录无法进行，从而影响肿瘤的发生和进展^[1-3]^。研究^[4-6]^显示，*SMARCA4*基因突变可在多种肿瘤中出现，包括肺癌、子宫肉瘤、卵巢高血钙型小细胞癌、鼻腔/鼻窦的未分化癌、胃肠道多形性癌、伯基特淋巴瘤等。其中，在已知的非小细胞肺癌（non-small cell lung cancer, NSCLC）细胞系中，30%-35%有*SMARCA4*基因的缺失，并且在一小部分原发性肺癌中也发现了该基因的突变^[7, 8]^。已有的研究提示，5%-10%的NSCLC中存在SMARCA4的丢失^[9-12]^，这类患者生存期短，预后不良^[11, 13, 14]^。由于以往的研究对该类肿瘤的临床病理形态学分析较少，本研究将回顾性分析2020年1月-2022年3月上海长海医院诊断的9例SMARCA4缺失的NSCLC（SMARCA4-deficient NSCLC, SMARCA4-dNSCLC）的临床病理学特征、免疫组化表达谱及鉴别诊断，加深对该病的认识。

## 1 材料与方法

### 1.1 病例资料

收集上海长海医院2020年1月-2022年3月诊断的9例SMARCA4-dNSCLC的临床资料，包括患者基本信息、吸烟史、临床症状、肿瘤原发灶-淋巴结-转移（tumor-node-metastasis, TNM）分期及临床分期、影像及随访，数据均由本院病例数据库及电话咨询获得。

### 1.2 免疫组织化学染色及判读方法

所有标本均经10%福尔马林充分固定，脱水、石蜡包埋、3 μm厚切片、苏木精-伊红（hematoxylin-eosin staining, HE）染色并观察其形态学特征。免疫组织化学采用EnVision两步法。所用一抗包括细胞角蛋白5.2（cytokeratin 5.2, CAM5.2）、细胞角蛋白7（cytokeratin 7, CK7）、甲状腺转录因子1（thyroid transcription factor-1, TTF-1）、p40、SMARCA4/Brg1（克隆号E8V5B），均为即用型工作液，除SMARCA4/Brg1购于北京中杉金桥公司外，其余抗体均购自福州迈新公司。二抗鼠/兔通用/即用型抗体购于北京中杉金桥公司。具体操作步骤严格按照试剂盒说明书进行。CAM5.2、CK7染色定位于细胞质，TTF-1、p40、SMARCA4染色均定位于细胞核。以SMARCA4在肿瘤细胞核染色强度显著减弱或完全缺失时判读为阴性，阳性对照为周围肺泡上皮或间质。

### 1.3 随访

所有病例均通过电话进行随访，截至2022年3月25日，生存期为手术日到死亡日或随访当日。

## 2 结果

### 2.1 临床资料

患者年龄43岁-74岁，中位年龄65岁。男性7例，女性2例。6例有吸烟史。3例肿瘤位于左上叶，1例左下叶，5例右上叶。9例中4例有咳嗽咳痰，其中1例伴有胸闷胸痛，1例伴有发热、胸水，5例无明显症状及体征。9例中4例因出现明显的临床症状就诊，1例因食管癌就诊查体发现肺部占位，4例因体检发现胸肺部肿块就诊。肿瘤大小1.0 cm-5.8 cm，平均直径3.3 cm。4例淋巴结转移，1例胸膜转移，1例肝脏转移。4例手术治疗，2例化疗，1例放疗，1例化疗联合免疫及局部放疗，1例转外院治疗。7例影像学提示为肺内边界不清的实性占位影，3例提示胸膜侵犯。病理及临床特征见表 1。

**表 1:** SMARCA4-dNSCLC的临床病理学特征 Clinicopathologic features of the SMARCA4-dNSCLC

No.	Site of the mass	Gross type	Symptoms	Smoking status	Pathology/imaging tumor size	Chief complaint	Biopsy type/site	Gender	Age(yr)	Metastasis	pTNM	Clinical staging	Treatment	Follow-up
1	Upper lobe of right lung	Peripheral type	Cough, expectoration	Unknown	2.4 cm	Cough	Bronchoscopy biopsy	Male	65	No lymph node dissection	T1cNxMx	IA3	Unknow	Lost to follow-up
2	Lower lobe of left lung	Peripheral type	No obvious symptoms	No	4 cm	Physical examinationfound tumor	Lobectomy specimen	Female	74	Lymph node metastasis	T2aN1Mx	IIB	Surgery	15 mon
3	Upper lobe of left lung	Peripheral type	Noobvious symptoms	Yes	3 cm	Physical examinationfound tumor	Wedge lung resection specimen	Male	69	Lymph node metastasis combined with liver metastases	pT1cNxM1	IVB	Chemotherapy	The patient died 15 mon after surgery
4	Upper lobe of right lung	Peripheral type	Cough, expectoration, chest tightness, chest pain	Yes	2.9 cm	Chest tightness, chest pain	Percutaneous lung puncture	Male	58	No lymph node dissection, pleural metastasis	pT2NxMx	IVA	Radiationtherapy	18 mon
5	Upper lobe of right lung	Centraltype	Cough, expectoration, mediastinal lymph node enlargement	Yes	5.4 cm	Cough, expectoration	11R lymph nodes	Male	69	Lymph node metastasis	pT3N2Mx	IIIB	Chemotherapy, immunotherapy, radiation therapy	9 mon
6	Upper lobe of right lung	Centralype	Pleural effusion with infection, pleural effusion, cough, expectoration, lymph node enlargement	Yes	5.8 cm	Cough, expectoration, fever, pleural effusion, lymph node enlargement	Bronchoscopy biopsy	Male	43	Lymph node metastasis	pT3N2Mx	IIIB	Chemotherapy	1 mon
7	Upper lobe of right lung	Peripheral type	No obvious symptoms	Yes	2 cm	Come to hospital for esophageal cancer and finds lung cancer	Wedge lung resection specimen	Male	67	No lymph node dissection	pT1bNxMx	IA2	Surgery	2 mon
8	Upper lobe of left lung	Peripheral type	No obvious symptoms	No	2.8 cm	Physical examinationfound tumor	Lobectomy specimen	Female	52	Lymph node metastases unclear	pT1cNxMx	IA3	Surgery	1 mon
9	Upper lobe of left lung	Peripheral type	No obvious symptoms	Yes	1 cm	Physical examinationfound tumor	Wedge lung resection specimen	Male	62	Lymph node metastases unclear	pT1aNxMx	IA1	Surgery	12 mon
SMARCA4-dNSCLC: SMARCA4-deficient non-small cell lung cancer; pTNM: pathological tumor-node-metastasis.														

### 2.2 病理形态学特征

镜下观察：9例SMARCA4-dNSCLC病理学形态大致为3种。①形态学为经典肺腺癌，肿瘤细胞立方形、矮柱状，不规则形，排列成不同分化程度的腺泡状、乳头状、微乳头状、实性巢片状，部分区域间质炎细胞浸润明显伴片状坏死。未见明确贴壁生长样结构（图 1）；②形态学为肺黏液腺癌，肿瘤细胞立方形，矮柱状，可见细胞外/内黏液，肿瘤组织排列成不同分化程度的腺泡状，乳头状、微乳头状及实性巢片状结构，间质可见不同程度的炎细胞浸润（图 2）；③形态学为低分化癌。肿瘤细胞为上皮样或合体样，胞质丰富，呈透明样或嗜酸性，部分细胞呈横纹肌样或未分化，局灶细胞梭形或肉瘤样，异型明显，部分核仁清楚，可见双核、多核细胞，染色质空泡状，核分裂象常见，排列成片巢状或结节状，可见嗜酸性小球，间质可见较多炎细胞浸润及片状坏死（图 3）。

**图 1:**
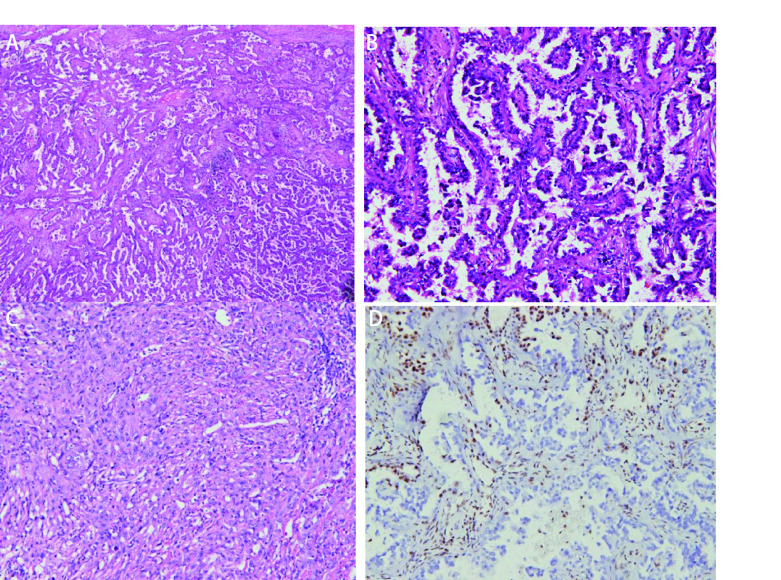
SMARCA4缺失的经典肺腺癌。A：经典肺腺癌，腺泡样、乳头状、微乳头状区域（HE染色, ×100）；B：微乳头区域（HE染色, ×200）；C：实体型、肉瘤样区域（HE染色, ×200）；D：SMARCA4阴性（免疫组化染色，×200）。 SMARCA4 deficient classic lung adenocarcinoma. A: Classic lung adenocarcinoma, acinar structure, papillary structure, micropapillary structure (HE staining, ×100); B: Micropapillary structure (HE staining, ×200); C: Solid structure, sarcomatoid structure (HE staining, ×200); D: SMARCA4 (-) (immunohistochemistry staining, ×200). HE: hematoxylin-eosin staining.

**图 2:**
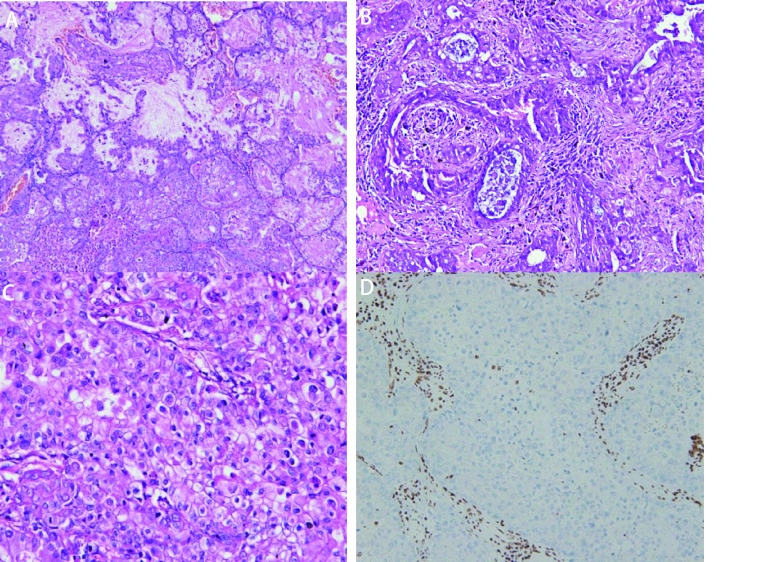
SMARCA4缺失的肺黏液腺癌。A：中至低分化黏液腺癌过渡区域（HE染色, ×200）；B：分化不良的腺样结构（HE染色, ×200）；C：实体型黏液腺癌（HE染色, ×200）；D：SMARCA4阴性（免疫组化染色，×200）。 SMARCA4 deficient mucinous adenocarcinoma of lung. A: Moderate to poor differentiated mucinous adenocarcinoma (HE staining, ×200); B: Poorly differentiated glandular structure (HE staining, ×200); C: Solid mucinous adenocarcinoma (HE staining, ×200); D: SMARCA4 (-) (immunohistochemistry staining, ×200).

**图 3:**
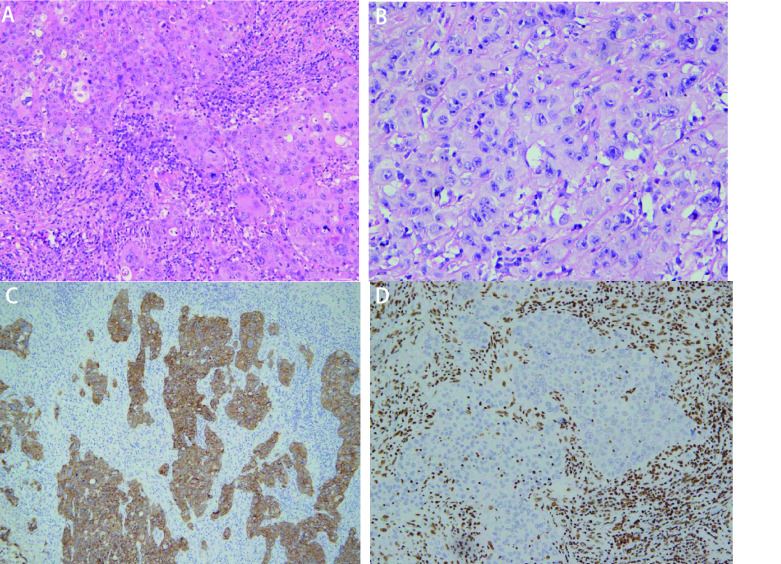
SMARCA4缺失的低分化癌。A：低分化癌。肿瘤细胞上皮样、合体样，呈片状，炎性间质（HE染色, ×200）；B：（HE染色, ×400）；C：CAM5.2阳性（免疫组化染色，×200）；D：SMARCA4阴性（免疫组化染色，×200）。 SMARCA4 deficient poorly differentiated carcinoma. A: Poorly differentiated carcinoma. The tumour cells showing epithelioid or syncytic and inflammatory cells in the stroma (HE staining, ×200); B: (HE staining, ×400); C: Tumor cells were diffuse positive for CAM5.2 (immunohistochemistry staining, ×200); D: Tumor cells showed SMARCA4 (-) (immunohistochemistry staining, ×200).

本组病例形态学上：2例表现为经典的非黏液性肺腺癌；2例为中至低分化黏液腺癌；5例为分化较差的低分化癌形态。9例均诊断为SMARCA4-dNSCLC，其中1例伴有肉瘤样分化，1例部分区域合并普通型肺腺癌。

### 2.3 免疫组织化学染色结果

8例肿瘤细胞低分子细胞角蛋白CAM5.2及CK7弥漫强阳性，1例肿瘤细胞CAM5.2、CK7部分区域强阳性，肉瘤样变区域弱阳性；天冬氨酸蛋白酶A（novel aspartie proteinase A, Napsin A）1例阳性，1例部分阳性，7例阴性；TTF-1 1例阳性，2例部分阳性，6例阴性；肺鳞癌标志物p40均阴性；增殖标志物Ki-67 5%-80%；SMARCA4蛋白2例部分阳性，7例阴性。结果见表 2。

**表 2:** SMARCA4-dNSCLC的免疫组化结果 Immunohistochemical results of SMARCA4-dNSCLC

No.	CAM5.2	CK7	Napsin A	TTF-1	P40	Ki67	SMARCA4
1	+	+	-	-	-	80%	-
2	+	+	-	-	-	40%	-
3	+	+	-	-	-	80%	-
4	+	+	-	-	-	80%	-
5	+	+	-	-	-	20%	-
6	+	+	-	-	-	25%	-
7	+	+	20%+	30%+	-	80%	20%+
8	+	+	+	+	-	5%	60%+
9	+	+	-	50%+	-	30%	-
CAM5.2: cytokeratin 5.2; CK7: cytokeratin 7; TTF-1: thyroid transcription factor-1; Napsin A: novel aspartie proteinase A.							

### 2.4 随访

截至2022年3月25日，对以上患者进行随访，1例失访，1例于治疗后15个月死亡。其余7例患者存活，随访时间1个月-18个月，中位随访时间9个月。见表 1。

## 3 讨论

### 3.1 SMARCA4与NSCLC

肺癌是我国最常见的恶性肿瘤，其中NSCLC占比高达85%，虽已有多种手段可用于晚期肺癌患者的治疗，但5年生存率仍较低。研究^[15]^表明，肺癌的发生不仅与致癌因素有关，还与非致癌因素或抑癌基因突变有关。目前研究较多的抑癌基因包括*SMARCA4*、*TP53*、*STK11*、*RB1*、*BRCA1*/*2*、*CDKN2A*等。其中在NSCLC中，*SMARCA4*/*BRG1*和*SMARCA2*/*BRM*基因的双重失活很常见，可引起SWI/SNF染色质重塑复合物中ATP酶的失活，从而诱导肿瘤细胞去分化，包括正常到低级别肿瘤转化，再到具有或不具有横纹肌特征的高侵袭性肿瘤转化^[16]^。2017年，Agaimy等^[11]^首次提出SMARCA4-dNSCLC是一类具有独特形态学特征、免疫表型及分子遗传学特征的罕见NSCLC，预后较差。随后，陆续有少量个案或小系列病例报道^[12, 17]^，多数报道提示该类肿瘤形态学常表现为低分化的NSCLC。综合文献复习和本组病例，我们发现SMARCA4-dNSCLC的病理学形态特征复杂多样，除了最常见的低分化肿瘤外，还有少量其他形态，在临床工作中需要警惕。

### 3.2 SMARCA4-dNSCLC的临床病理学特征

多项研究^[11, 12]^提示，SMARCA4-dNSCLC常见于吸烟中老年男性患者，是一类具有明确病理学特征的侵袭性肺癌，肿瘤体积较大，常伴胸膜或脉管侵犯，总生存期短。本组9例中7例为男性患者，中位年龄65岁，6例有吸烟史，7例影像学呈边界不清的侵袭性特征，肿瘤平均直径3.3 cm，3例可见胸膜侵犯，5例手术切除的标本中3例可见脉管癌栓，5例诊断时已转移，这些特点与上述研究结果类似。本组9例患者1例术后15个月死亡，1例失访，7例生存，随访1个月-18个月，中位随访时间9个月，虽多数患者生存，但随访时间较短，尚无法明确这类肿瘤的预后情况。

在组织学上，文献^[10-12, 16-19]^报道SMARCA4-dNSCLC的病理学形态可从分化较好的肺腺癌到差分化腺癌甚至分化不良的恶性肿瘤，其中大多数病例表现为低分化癌。本组研究观察到肿瘤组织可表现为3种形态学特征：①大多数病例表现为低分化或去分化形态，细胞上皮样或合体样，横纹肌样，甚至梭形或肉瘤样，胞质可完全透明至嗜酸性，可见嗜酸性小球或小脓肿，间质较多炎细胞浸润及片状坏死^[11, 12]^，本组7例病例可观察到这种形态；②少见的黏液腺癌。Agaimy等^[11]^首次在20例SMARCA4-dNSCLC中发现2例为黏液腺癌，随后陆续有少量文献^[16, 17]^报道这一形态，本组观察到2例黏液腺癌，其中1例同Agaimy等^[11]^报道的形态一致，均为不规则腺管为主的中低分化的黏液腺癌；另一例为中分化黏液腺癌伴低分化实体型腺癌且只有低分化区域SMARCA4缺失，这与Naito等^[12]^报道一致，提示中分化肺腺癌可与分化差的区域并存，且随着SMARCA4的缺失，肿瘤组织去分化而发生进展；③本组病例中2例表现为经典的肺腺癌结构，结合既往文献^[10, 17]^报道，SMARCA4缺失的肿瘤组织可呈腺泡状、乳头状结构，但未见微乳头结构。本组1例在部分微乳头结构区观察到SMARCA4表达缺失，提示SMARCA4缺失的病例也可表现为微乳头结构。总之，对于NSCLC，在伴不同程度的上皮分化同时有明显的炎性间质背景和坏死时，应考虑到SMARCA4-dNSCLC的可能；另外除贴壁生长型区域外，SMARCA4-dNSCLC可表现为多种形态学特征，包括腺管样结构、乳头样结构、微乳头样结构及实体型结构，且随着SMARCA4蛋白的进一步缺失，肿瘤组织进一步去分化甚至出现肉瘤样变。

### 3.3 SMARCA4-dNSCLC的免疫组化谱

这类肿瘤SMARCA4常缺失或弥漫性显著减弱；上皮标志物pCK、CK7、HepPar1、CAM5.2、CK7呈弥漫强阳性。肺腺癌标志物TTF-1在80%-90%病例中为阴性，少数病例中为弱-中等表达^[10, 11, 18]^，鳞癌标志物p40均为阴性。本组病例8例CAM5.2与CK7均弥漫强阳性，可与SMARCA4-缺失的未分化肿瘤（SMARCA4-deficient undifferentiated tumor, SMARCA4-DUT）鉴别，1例中低分化的病例CAM5.2与CK7部分区域强阳性，肉瘤样变区域表达弱阳性；TTF-1阴性6例，完全阳性1例，部分阳性2例。我们发现，本组2例TTF-1部分阳性的病例在肿瘤组织低分化区域甚至肉瘤样变区域失表达，同时SMARCA4在对应的低分化区域表达亦呈逐渐减弱趋势甚至失表达，而高中分化区域TTF-1及SMARCA4正常表达，这与Yoshida等^[19]^的研究结果类似，猜测可能由于SMARCA4的缺失促进了肿瘤组织的去分化，而SMARCA4-dNSCLC是否可进一步去分化转化为SMARCA4-DUT，有待后续进一步分析。

### 3.4 鉴别诊断

需要鉴别的肿瘤包括：①经典的肺腺癌：有研究^[20]^在观察了146例肺腺癌标本后发现5.5%的病例存在SMARCA4的缺失，腺泡状、乳头状亚型尤其是伴有实体型成分的病例均不能除外SMARCA4-dNSCLC的可能，建议在诊断时加做SMARCA4的免疫组化以资鉴别。普通型肺腺癌SMARCA4、TTF-1阳性，SMARCA4-dNSCLC中TTF-1多数阴性，SMARCA4至少部分缺失表达；②SMARCA4-DUT：这是2021版世界卫生组织（World Health Organization, WHO）胸肺分册新提出的一种高度恶性的未分化肿瘤，常见于纵隔、肺门或肺，而SMARCA4-dNSCLC常发生于肺实质。差分化的SMARCA4-dNSCLC需与之鉴别。这类肿瘤细胞大而圆，上皮样，可见横纹肌样细胞，核分裂象及坏死常见，大多数病例无明确的上皮分化区域，而SMARCA4-dNSCLC可见不同程度的上皮分化^[21]^。免疫组化两者SMARCA4均缺失，但SMARCA4-DUT缺乏或局灶表达上皮标志物pCK、CK7、CAM5.2，而SMARCA4-dNSCLC弥漫强阳性表达；SMARCA4-DUT干细胞标志物SALL4、SOX2、CD34局灶或弥漫阳性表达，SMARCA4-dNSCLC无表达^[22]^；③神经内分泌肿瘤：这类肿瘤分化差时需与本病鉴别，但其SMARCA4阳性，且上皮标志物pCK与神经内分泌标志物CgA、Syn、CD56常阳性，但SMARCA4-dNSCLC神经内分泌标志物及SMARCA4蛋白通常阴性；④上皮样间皮瘤：Yoshikawa等^[23]^的研究显示在部分上皮样间皮瘤中也存在SMARCA4的缺失。这类病例可见上皮样的肿瘤组织呈腺样、乳头样结构，与经典肺腺癌类似，但免疫组化显示间皮标志物WT-1/D2-40/Calretinin/CK5/6常阳性表达，可资鉴别；⑤其他可发生于肺部的差分化肿瘤：包括Nut癌、淋巴瘤、生殖细胞肿瘤、黑色素瘤、各种差分化肉瘤均需鉴别^[24]^。可通过免疫组化谱NUT、LCA、SALL4、Lin28、S100、HMB45、MyD1、Myogenin等标志物进行鉴别；⑥其他转移性SWI/SNF复合体异常的肿瘤：由于肺是全身多种肿瘤的常见转移部位，且SMARCA4缺失型肿瘤可见于多种组织器官，故诊断时应结合临床、影像及免疫组化结果，除外妇科、鼻咽部、消化道等部位的转移性肿瘤^[25]^。

### 3.5 基因检测手段

目前，二代测序（next generation sequencing, NGS）检测已经证明并不是所有的*SMARCA4*突变均可引起BRG1蛋白的缺失，单纯的错义突变或单个等位基因的突变，并不会导致BRG1蛋白的缺失，只有通过无意或框内缺失的双等位基因的突变才会引起蛋白表达的缺失^[11, 14]^。在实际工作中，大多数病例可以通过免疫组化检测来判断BRG1蛋白的缺失，对于部分无法明确的病例，推荐进行分子检测^[20]^。由于*SMARCA4*不是热点突变，它在整个基因中有多个突变。因此，需要对全基因组进行测序。已确定了9个*SMARCA4*基因突变，其中包括Glu1023^*^和Glu1346的删除突变以及肺腺癌患者常见的错义突变G1232S/V^[12]^。同时，NGS发现SMARCA4-dNSCLC涉及到的驱动基因突变主要有*SMARCA4*、*p53*、*KRAS*、*STK11*、*KEAP1*、*NF1*，而与经典肺腺癌的驱动基因表皮生长因子受体（epidermal growth factor receptor, *EGFR*）、间变性淋巴瘤激酶（anaplastic lymphoma kinase, *ALK*）、*ROS1*不相关^[11]^。总之，通过免疫组化方法即可对SMARCA4-dNSCLC做出病理诊断，NGS可同时识别所有癌基因和抑癌基因，为进一步阐释其致病机制提供依据。

### 3.6 SMARCA4-dNSCLC的临床意义

由于SMARCA4-dNSCLC是近来新提出的肿瘤分类，临床上目前还没有统一的标准化治疗方案。但已有的诸多前期临床研究对该类肿瘤的治疗提供了新的契机，包括调控表观遗传学的药物[组蛋白去乙酰化酶（histone deacetylase, HDAC）抑制剂、组蛋白修饰剂或组蛋白去甲基化酶]、溴结构域和末端外基序蛋白抑制剂（bromodomain and extra-terminal domain inhibitor, BETi）、铂类化疗药物以及免疫检查点抑制剂（immune checkpoint inhibitors, ICIs）等。其中ICIs的应用在临床上改变了传统的NSCLC的治疗模式，显示出越来越重要的地位。研究^[12, 14]^提示，NSCLC中SMARCA4的缺失与程序性细胞死亡配体1（programmed cell death ligand 1, PD-L1）的阳性状态及较高的肿瘤突变负荷（tumor mutational burden, TMB）相关。这为SMARCA4-dNSCLC患者的免疫治疗提供了新的依据。一项关于免疫治疗效果的研究^[26]^显示，在445例接受了ICIs治疗的患者中，*SMARCA4*突变的患者较SMARCA4野生型患者具有更高的客观缓解率及生存率，提示*SMARCA4*突变型肺癌对免疫治疗的敏感性更高。另一例43岁的SMARCA4-dNSCLC的吸烟男性患者，在使用标准化疗方案后出现多发肺转移和复发，全外显子测序显示，PD-L1失表达，但TMB高。临床使用四线纳武利尤单抗治疗后，疾病控制达14个月以上^[12]^。提示ICIs的应用可能是SMARCA4-dNSCLC最有前景的治疗方案之一。

### 3.7 总结

SMARCA4-dNSCLC的组织病理学特点复杂多变，这为临床工作中准确识别该类肿瘤带来了较大的挑战。实际工作中对于中低分化的NSCLC建议加做SMARCA4免疫组化，除外SMARCA4-dNSCLC的可能。由于这类肿瘤目前还没有统一的标准化治疗方案，只有先做出准确的病理诊断，才能为后续制定该类肿瘤的治疗策略提供新的思路。
